# Complex Internal Microstructure of Feather Follicles on Chicken Skin Promotes the Bacterial Cross-Contamination of Carcasses During the Slaughtering Process

**DOI:** 10.3389/fmicb.2020.571913

**Published:** 2020-09-17

**Authors:** Xibin Zhang, Zixin Peng, Peng Li, Yanwei Mao, Ru Shen, Rui Tao, Xiuguo Diao, Longhai Liu, Yuzhong Zhao, Xin Luo

**Affiliations:** ^1^College of Food Science and Engineering, Shandong Agricultural University, Tai’an, China; ^2^New Hope Liuhe Co., Ltd., Laboratory of Feed and Livestock and Poultry Products Quality & Safety Control, Ministry of Agriculture, Beijing, China; ^3^Key Laboratory of Food Safety Risk Assessment, Ministry of Health, China National Center for Food Safety Risk Assessment, Beijing, China; ^4^Department of Food Science and Engineering, Qingdao Agricultural University, Qingdao, China

**Keywords:** feather follicles, HE staining, closed cavity, biopsy sampling, cross-contamination

## Abstract

Chicken skin is considered the most susceptible to bacterial contamination during slaughter. It is rich in bushy feather follicles with complex internal structures that can absorb bacteria via cross-contamination during slaughter. Until now, the microstructural changes and local bacterial composition of feather follicles during slaughter have not been thoroughly investigated. This study used hematoxylin-eosin (HE) staining of the tissue paraffin section to investigate the structure of the feather follicles on chicken skin. In addition, the biopsy sampling method was employed for the high-throughput sequencing of 16S RNA genes to study the composition and source of bacterial contamination during slaughter. The results show that the feather follicles on chicken skin form a closed cavity structure during the slaughtering process. The volume of the irregular follicle cavity was about Ø: 200 μm × D: 1040 μm, which provides a place for the bacteria to absorb and resist the cleaning and disinfection during the slaughtering process. The composition of bacteria in the feather follicle was mainly *Acinetobacter* (37%), *Psychrobacter* (8%), *Macrococcus* (5%), and *Comamonas* (2%). The heat map obtained via the species abundance analysis of the feather follicle samples as well as the slaughter environment samples suggests that the gastrointestinal feces contaminated the feather follicles on the chicken skin mainly during the evisceration, defeathering, and chilling processes, and the last-stage chilling water also caused severe cross-contamination to the feather follicles during the chilling process.

## Introduction

Chicken has become a widely consumed meat worldwide due to its rich nutrition, abundant supply, and low cost (OECD, 2013). However, fresh chicken is susceptible to spoilage-related microbial group contamination, such as ephemeral/specific spoilage organisms (E/SSO), destroying the shelf life of products and resulting in massive economic losses (Doulgeraki et al., 2012; Wang et al., 2017). Furthermore, it is generally believed that the complex structure of chicken skin tissue makes it more susceptible to bacterial contamination because it is difficult to clean and reduce bacteria during slaughter (Berndtson et al., 1992; Yang et al., 2001; Cason et al., 2004; Jang et al., 2007; Latt et al., 2018).

Many studies have documented the location of contamination bacteria on the surface microstructure of chicken skin as well as bacterial cross-contamination during slaughter (Cason et al., 2004; Chantarapanont et al., 2004; Jang et al., 2007; Latt et al., 2018). Jang et al. (2007) find that the adsorption sites of *Campylobacter jejuni* are located on the skin surface and in the follicles.

Many studies document the bacterial pollution routes and compositions during the slaughter process of chickens with the help of high-throughput 16S rRNA sequencing technology, which overcomes the unculturable and laborious limitations. The dominant bacteria on the carcass skin after chilling primarily include *Proteobacteria*, *Firmicutes*, *Bacteroidetes*, and *Actinobacteria* at the phyla level and *Acinetobacter, Pseudomonas*, *Lactobacillus*, *Streptococcus*, *Staphylococcus*, and *Chryseobacterium* at the genus level (Zweifel et al., 2015; Samapundo et al., 2019; Wang et al., 2019; Chen et al., 2020). The scalding, defeathering, evisceration, and chilling processes were identified as responsible for substantial cross-contamination during typically automated chicken slaughter (Chen et al., 2020). Furthermore, HE staining is a mature histopathological method used for visually observing the inner microstructural changes of tissues (Hussein et al., 2019). Mini punch is a tissue transplantation and biopsy sampling method widely used during medical skin surgery (Huang et al., 2012; Hirobe and Enami, 2018; Fofanov et al., 2019), and it can be combined with other analytical methods to realize the detection and analysis of trace samples.

Up to now, the subcutaneous structure of the follicles as well as the bacterial community *in situ* have not been fully revealed, impeding the development of new technology for reducing bacterial contamination in the poultry industry. This study analyzes the internal microstructure and the local bacterial composition of feather follicles using HE staining and circumferential scouting punch biopsies to explore the role of the follicle in the cross-contamination of the skin and to pave the way for implementing feasible contamination-reduction measures during the commercial slaughter process.

## Materials and Methods

### Sample Collection and Preparation

Seventeen types of high-throughput 16S rRNA sequencing samples (Table 1), including chicken skin and follicles, carcass appendages, and the water and surface dirt of the facilities, were collected continuously during the slaughtering process. The skin and feather follicle samples were uniformly obtained via Ø3-mm sterile circumferential scouting punches (Miltex, United States) from five different parts of the chicken carcass, which were collected randomly during the four key processing steps. In addition, the broiler involved in this study, a white feather Ross 308 cultured for 36 days, came from the same farms with the same batches of chicks, feed, and breeding conditions. The samples of chicken skin with feather follicles were named SAB (after bleeding), SAD (after defeathering), SAE (after evisceration), and SAC (after chilling).

**TABLE 1:** The sampling scheme for bacterial contamination of chicken skin follicles during slaughter.

**Sample category**	**Bleeding**	**Scalding and defeathering**	**Evisceration**	**Chilling**	**Cutting**
Carcass rinse	\	CRAD	CRAE	CRAC	\
HE staining skin	SAB	SAD	SAE	SAC	\
Chicken follicles	SAB	SAD	SAE	SAC	\
Carcass appendages	AA, AC, AF	\	\	\	\
Facilities water	\	FWS	\	FWCF, FWCM, and FWCL	\
Facilities surface dirt	\	FDF	FDE	FDCF, FDCM, and FDCL	FDBC

The samples of the carcass appendages were collected evenly from the slaughter line. Sterile scissors were used to obtain the AF (feather) sample, and the AC (craw contents) and AA (anal contents) were collected with cotton swabs. Water samples from the slaughter facilities: scalding tank (FWS) and the first, middle, and last chiller (FWCF, FWCM, and FWCL) were collected using a sterile container and membrane filter (Merck EZ-Fit Filtration Unit, Germany). The surface dirt samples of the slaughter facilities: the conveyor belt in the cutting area (FDBC); the rubber fingers in the defeathering machine (FDF); evisceration (FDE); and the first, middle, and latter chiller underwater walls (FDCF, FDCM, and FDCL) were randomly obtained using a sterile cotton swab and metal scraper. All samples were rapidly frozen using liquid nitrogen and transported in drikold to the genome-sequencing lab.

The chicken carcass rinse samples were collected along the processing line following sterile sampling technique requirements and included CRAD (after defeathering), CRAE (after evisceration), and CRAC (after chilling). Three carcasses from the same broiler flock were collected randomly at each key processing site and subjected to a whole carcass rinse by adding 400 ml sterile buffered peptone water (BPW). They were then thoroughly agitated by hand for 120 s to ensure that the surface as well as the internal and external parts of the birds came in full contact with the BPW in a sterile stomacher bag.

### Microbiological Analysis

After gradient dilution, all rinse samples of the chicken carcasses were plated onto PetriFilm Aerobic and Coliforms (COLI) count plates (3M, United States) to detect the total viable count (TVC) and COLI. These two bacterial indicators were enumerated and logarithmically transformed as total colony-forming units per ml (Lg10 CFU/ml).

### The Microstructure of the Follicles Obtained via Paraffin HE Staining and CAD

Three random breast skin samples were collected from the broilers during each of the three key processing stages, namely SAB, SAD, and SAC. Representative skin tissue samples of 3^∗^3 cm were obtained from the same position on the chest of the carcass using sterile scissors and forceps. All the collected HE stained skin samples were fixed directly with 10% (v/v) neutral formaldehyde. Tissue sections were prepared, deparaffinized, stained with HE, and then observed and photographed using light microscopy. The scale function of Auto CAD 2018 software was used to measure the size of typical feather follicle structures in the paraffin sections of chicken skin.

### Next-Generation Sequencing (NGS) and Data Analysis

#### Extraction of Genomic DNA and Amplicon Generation

The total genomic DNA was extracted from the samples using the cetyltrimethylammonium bromide (CTAB) method. Purified genomic DNA was monitored on 1% (w/w) agarose gel. According to the concentration, DNA was diluted to l ng/pL using sterile water, after which the16S rRNA genes of particular regions (16S V3–V4) were amplified using specific primers (16S V3+V4; 341F: CCTAYGGGRBGCASCAG, 806R: GGACTACHVGGGTWTCTAAT) with a barcode. All PCR reactions were performed with 15 pL of Phusion^®^ High-Fidelity PCR Master Mix (New England Biolabs, US), 0.2 μM of forward and reverse primers, and about 10 ng template DNA. The thermal cycling consisted of initial denaturation at 98°C for 1 min, followed by 30 cycles of denaturation at 98°C for 10 s, annealing at 50°C for 30 s, elongation at 72°C for 30 s, and finally, 72°C for 5 min. The same volume of 1X loading buffer (containing SYB green) was mixed with the PCR products and subjected to electrophoresis on 2% agarose gel for detection. The PCR products were combined at equal ratios, after which they were purified with a Qiagen Gel Extraction Kit (Qiagen, Germany).

#### Generating Libraries and NGS

Sequencing libraries were generated using a TruSeq^®^ DNA PCR-Free Sample Preparation Kit (Illumina, US) following the manufacturer’s recommendations, after which index codes were added. The library quality was assessed on the Qubit@2.0 Fluorometer (Thermo Fisher Scientific, United States) and Agilent Bioanalyzer 2100 system (Agilent, United States). Finally, the library was sequenced on an Illumina NovaSeq platform (Illumina, United States), generating 250 bp paired-end reads.

### Data Analysis

#### Operational Taxonomic Unit (OTU) Clustering and Species Annotation

The sequences analysis was performed using Uparse software (Uparse v7.0.1001) (Edgar, 2013), and sequences with >97% similarity were assigned to the same OTUs. Representative sequences for each OTU were screened for further annotation. For each representative sequence, the Silva Database (Christian et al., 2012) was used based on the Mothur algorithm to annotate the taxonomic information. Furthermore, to examine the phylogenetic relationship between different OTUs and the differences between the dominant species in various samples (groups), multiple sequence alignments were conducted using the MUSCLE (MUltiple Sequence Comparison by Log- Expectation) software (Version 3.8.31) (Edgar, 2004). The information regarding the OTU abundance was normalized using a standard sequence number corresponding to the sample with the least sequences. Subsequent analysis of alpha and beta diversity were all performed based on this normalized output data. The heat map of the top 35 genera in all the sampling groups in chicken slaughter was generated using Euclidean distance and complete linkage algorithm implemented in the ggplot2 package of R software.

#### Diversity Analyses

Alpha diversity is applied in analyzing complexity of species diversity for a sample. The Observed-species in these samples was calculated with QIIME (Version 1.7.0) and displayed with R software (Version 2.15.3), and the Wilcox rank sum test was used to analyze whether the mean difference of species diversity between groups was significant.

### Statistical Analysis

All measurements were expressed as the mean ± standard error. Differences in the aerobic bacteria plate count and the COLI count of the skin during different processing stages were explored via the one-way Duncan’s ANOVA procedure using SPSS 19.0 at *P* < 0.05. A *T*-test was used to assess the beta species diversity between the groups and to determine the differences between these species.

## Results

### Bacterial Count Analysis of the Carcass Rinse During Three Key Processing Stages

As shown in Table 2, the TVC indexes of chicken carcass rinse were significantly decreased (*P* < 0.05) during the chilling stage from 4.56 to 3.56 lg CFU/ml although the COLI decreased from 3.96 to 2.37 lg CFU/ml. In addition, the evisceration process significantly increased the TVC of the carcass rinse (*P* < 0.05) from 4.56 to 5.19 CFU/ml while, interestingly, the COLI was not significantly changed (*P* > 0.05), which might show that the process of the defeathering step also occurred in the intestinal content contamination.

**TABLE 2:** The bacterial culture count of the chicken carcass rinse at three key processing points.

**Sample source**	**TVC Lg (CFU/ml)**	**COLI Lg (CFU/ml)**
CRAD	4.56 ± 0.29 b	3.86 ± 0.10 a
CRAE	5.19 ± 0.03 a	3.96 ± 0.20 a
CRAC	3.56 ± 0.13 c	2.37 ± 0.24 b

*Data are expressed as mean ± standard error, standard error = standard deviation/sqrt (n), n = 4. Data within the same column are not significantly different from each other when having the same letter. TVC, Total aerobic and mesophilic bacteria visible colony plate count; COLI, Coliform count.*

### Analysis of the Internal Morphology and Structure of the Feather Follicles During Slaughter

**FIGURE 1:**
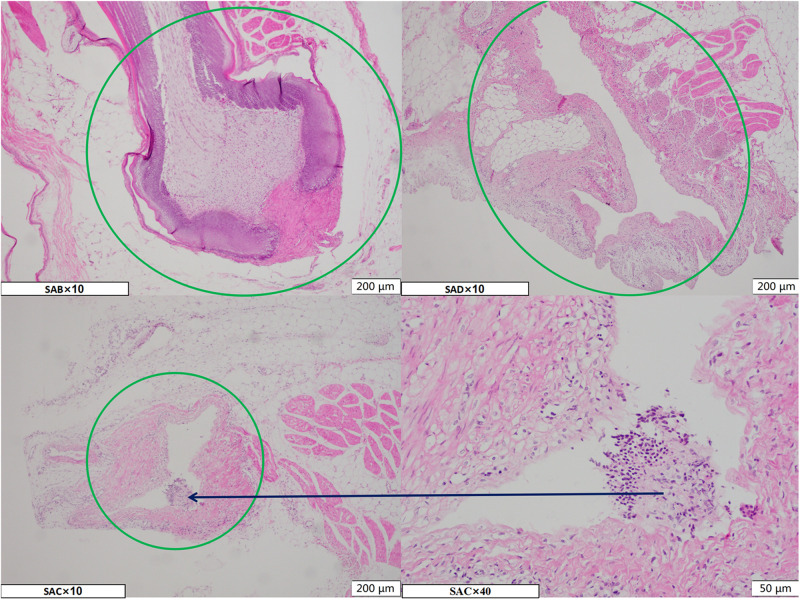
The microscope image of the HE staining paraffin section of the chicken breast skin during three processing stages. SAB, SAD, and SAC group indicate, respectively, samples of skin with feather follicles after bleeding, defeathering, and chilling processes. The green circle marks the area of the feather follicle, and the black arrows represent local magnification of the corresponding area.

The normal broiler breast skin feather follicle (SAB Ø: 403 μm ^∗^ D: 1955 μm) and after pulling the feather rod from the skin during the defeathering process results in an empty feather follicle (SAD Ø:200 μm^∗^D:1040 μm) in the skin, forming a closed internal cavity (SAC – Ø:100 μm^∗^D:560 μm) due to the contraction of the skin at the opening after chilling (Figure 1).

The follicle cavities were filled with fluid and contained feather fragments as well as dirt particles from the slaughterhouse. Consequently, the residue and debris caused bacterial cross-contamination in the feather follicle cavity. These phenomena indicate that the empty follicle cavity presents a negative pressure that could absorb the liquid on the skin surface of the carcass while subjected to the defeathering process.

### Microbiome Analysis

#### The α Diversity Analysis of the Follicles and Potential Contamination of Samples

**FIGURE 2:**
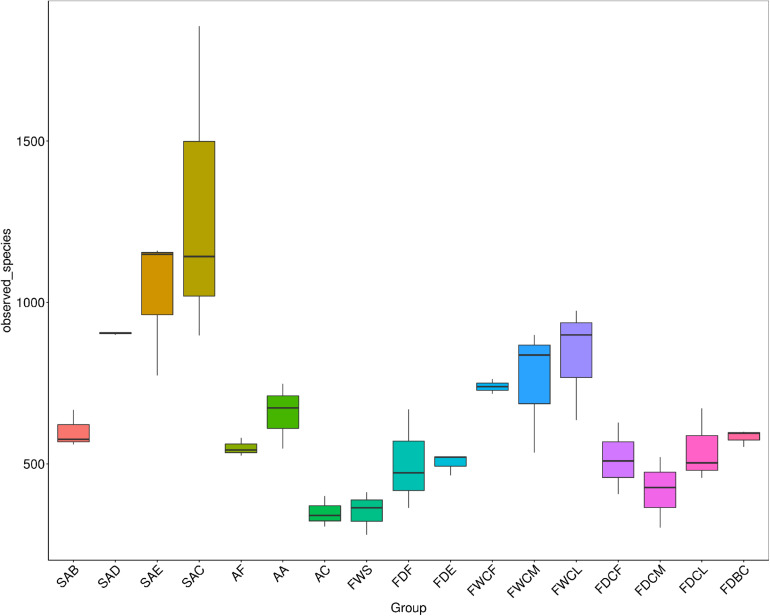
The analysis of the differences in the α diversity index between the follicles and potential contaminant samples during slaughter. SAB, SAD, SAE, and SAC group indicate, respectively, samples of skin with follicles after bleeding, defeathering, evisceration, and chilling processes. AF, AA, and AC group indicate, respectively, the carcass appendage samples of feather, craw, and anal contents. FWS, FWCF, FWCM, and FWCL group indicate, respectively, the water samples from the slaughter facilities: scalding tank and the first, middle, and last chillers. FDF, FDE, FDBC, FDCF, FDCM, and FDCL group indicate, respectively, the surface dirt samples of the slaughter facilities: defeathering; evisceration; the conveyor belt in the cutting area; and walls underwater of the first, middle, and latter chillers.

As shown in Figure 2, the bacterial species diversity of SAB, SAD, SAE, and SAC increased significantly with the continuation of the slaughter process (*P* < 0.05), and the diversity of the bacterial species in the follicles after defeathering was no less than that from a single source in a slaughterhouse, indicating that the bacterial species in the skin of the chicken carcass increased significantly due to cross-contamination during the slaughter process.

The bacterial diversity in the craw content of the chickens and the scalding water was the lowest, indicating that the composition of the bacterial species in these two regions was relatively simple. The diversity of bacteria in the surface dirt of the slaughtering facilities was similar with only that on the FDCM being slightly lower.

#### Bacterial Composition of the Follicles and Potential Contamination of Samples During Slaughter

**FIGURE 3:**
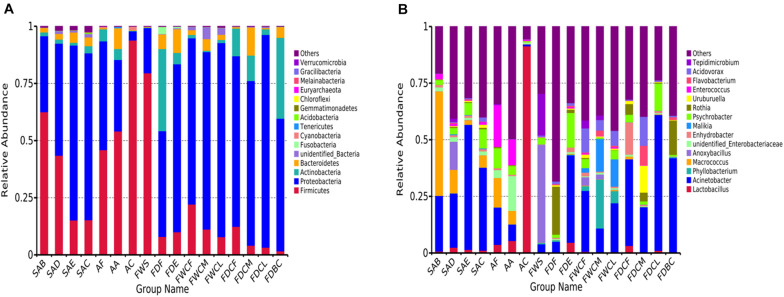
Relative abundance of top 15 bacterial composition at phyla **(A)** and genus **(B)** levels in the follicles with the potential to contaminate samples.

The analysis of the OTU clustering and the annotation information (Figure 3) indicates that *Proteobacteria* (58.1%), *Firmicutes* (28.9%), *Actinomycetes* (7.3%), and *Bacteroidetes* (4.0%) were the dominant bacteria in the feather follicle samples at the phyla level. During slaughter, the relative abundance of *Firmicutes* decreased, and the relative abundance of *Proteobacteria* increased and occupied the proportion of its vacancy. The *Firmicutes* levels in the carcass appendage samples, namely AF, AA, and AC, were significantly higher than *Proteobacteria.* However, the relative abundance of *Proteobacteria* was significantly higher than *Firmicutes* in the water and dirt samples of the slaughtering facilities.

The dominant bacterial genera exhibiting high abundance in the follicles after chilling at the genus level included *Acinetobacter* (36.6%), *Psychrobacter* (8.5%), *Macrococcus* (5.4%), *Aeromonas* (2.2%), *Comamonas* (2.0%), *Acidovorax* (1.9%), *unidentified Enterobacteriaceae* (1.7%), *Pseudomonas* (1.6%), *Arcobacter* (1.4%), *Uruburuella* (1.4%), *Kurthia* (1.3%), *Enterococcus* (1.2%), *Vitreoscilla* (1.0%), and *Enhydrobacter* (1.0%). These results were significantly different from the initial bacterial composition in the feather follicles with *Macrococcus* showing a significant decline, and *Acinetobacter* and *Psychrobacter* displaying a substantial increase. These findings suggest that the slaughter operation causes considerable changes in the structures of the bacterial communities in the chicken follicles.

#### Analysis of Follicle Contamination Sources via a Species Clustering Heat Map

**FIGURE 4:**
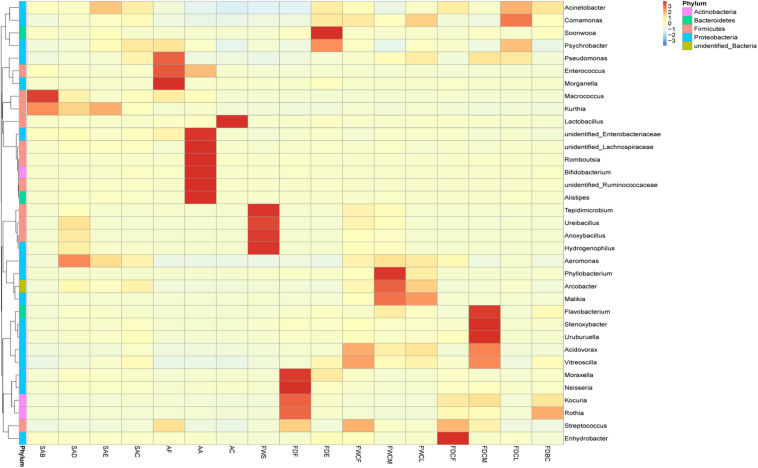
Heat map analysis of the relative species abundance clustering in the feather follicles and pollutants. The different colors indicate the species abundance, and a deeper color indicates a higher relative abundance in the sample.

The cluster analysis results of the relative bacterial abundance in the feather follicles and the environmental pollutants during the slaughter process are shown in Figure 4. Therefore, changes in the relative bacterial abundance in the feather follicles and the distribution of environmental pollutants during slaughtering as well as assessing the contact between the carcasses and these pollutants during the four critical processing stages facilitated the analysis of the bacterial contamination sources. The dominant bacteria before chicken skin depilation were *Macrococcus* and *Kurthia*, and their relative abundance decreased significantly during depilation and subsequent processing, indicating that these two bacteria were native to the skin. The scalding and defeathering processes increased the relative abundance of *Aeromonas*, *Anoxybacillus* (*P* < 0.5), *Ureibacillus*, and *Tepidimicrobium*, and *Anoxybacillus*, *Ureibacillus*, and *Epidimicrobium* primarily originated from scalding water. *Aeromonas* might mainly come from the chicken’s craw during defeathering. During evisceration, *Acinetobacter* and *Psychrobacter* displayed a significant increase (*P* < 0.5) in the feather follicles. *Acinetobacter* might originate mainly from contents exposed to intestinal damage, and *Psychrobacter* might result from untreated running water used to rinse the carcass.

During the chilling and disinfection process, the relative abundance of *Acinetobacter* (55.2–36.6%) decreased (*P* > 0.05). However, the relative abundance of *Psychrobacter* (5.4–8.5%) and *Pseudomonas* (0.6–1.6%) was higher (*P* > 0.05). Except for *Phyllobacterium*, *Aeromonas*, etc., the relative abundance of other bacteria in the FDCM was the lowest of the three chilling-water levels and could mainly be attributed to the disinfectant effect of sodium hypochlorite. During the chilling process, the relative abundance of *Pseudomonas*, *Psychrobacter*, and *Comamonas* in the feather follicles displayed a significant increase, and the main sources of these SSO were the FDCM and FDCL of the facilities.

## Discussion

Defeathering caused an empty cavity to form in the chicken skin tissue that was susceptible to bacterial contamination, which gradually led to the closure of the cavity due to the contraction of the surface skin layer during chilling. This process sealed the follicle cavity of the follicles, promoting the resistance of the bacteria to disinfectants and cleaning. The paraffin sections of the different stages (Figure 1) indicate that a percentage of the follicles shrunk and closed the entrances with a fatty layer of skin due to post-mortem rigidity, which is a normal phenomenon in dead animal tissues (Barbut, 2015; Latt et al., 2018). These organic-rich tissue fluids in the follicles also provide protection for the absorbed bacteria against disinfection by consuming the disinfectant because feather plucking can injure the follicle’s primary tissue, allowing the tissue fluid to ooze out.

The folded structure and empty cavity inside the follicles could provide adsorption sites for bacterial contamination. The microstructure and surface of the feather follicle exhibit deformation that could be ascribed to relaxation when the chicken skin is exposed to hot scalding at 60–62°C, for 110 s and intense mechanical defeathering. Due to the feather being uprooted during the defeathering operation, subcutaneous tissue and muscle as well as the fat layer at the bottom of the endothelial cell are severely damaged, which results in more traumatic interfaces. After chilling, the opening of the closed feather follicle is blocked by the fatty layer of skin, which is also hydrophobic. Therefore, water and water-soluble disinfectant components are kept out by the constricted skin at the follicle outlet, preventing these compounds from penetrating the follicle. In addition, the organic matter in chicken skin also can come apart to wait for disinfectant-free chlorine (Yang et al., 2001). This result suggests that the structures of the feather follicles responsible for the closed cavities provide protection for contaminating bacteria inside against washing and disinfection.

Interestingly, environmental samples and carcass appendages from broiler slaughterhouses have a very characteristic bacterial composition; for example, *Lactobacillus* (91.4%) dominated in AC, and FWS was rich in spore-forming bacteria, such as *Anoxybacillus* (43.2%), *Epidimicrobium* (18.5%), and *Ureibacillus* (11.0%). These bacteria are often found in hot springs (Weon et al., 2007; Fritze and De Vos, 2009; Pikuta et al., 2015; Slobodkin et al., 2017), indicating that scalding water during regular production days is the closest to the unique microenvironment of hot springs during slaughter. *Psychrobacter* dominated in FDCL, which could be related to *Psychrobacter* contamination of the slaughter production water.

The feather follicles absorbed spoilage-related bacteria from the carcass appendages and water of the slaughter facilities after defeathering. Chilling water, initially used for cleaning and disinfecting the carcasses, is likely to become a source of spoilage bacteria in actual slaughterhouses, which can adversely affect the initial bacterial count on chicken skin. According to the analysis of the source of bacterial contamination in the follicle, the presence of *Acinetobacter* mainly resulted from evisceration and depilation, and *Pseudomonas* and *Psychrobacter* primarily originated during chilling and evisceration. Therefore, controlling bacterial contamination of the chicken skin at the source is essential. It is known that chicken are the natural host of *Campylobacter* (*jejuni* and *coli*) which are common foodborne pathogens worldwide, and *Campylobacter* jejuni has a high contamination rate during the slaughter of broilers (Barbut, 2015; Chen et al., 2020). However, the *Campylobacter* did not appear in the top 15 relative abundance genera of chicken skin with follicles and slaughter environmental samples in this study, and the same results were seen in another study of bacterial contamination during slaughtering of yellow-feathered chickens (Wang et al., 2019). This may be related to local farming practices and disease control measures.

When the follicle forms a closed cavity structure during the chilling process, it provides protection to the bacteria absorbed by follicle against various commonly used chemical bacteriostatic agents (Berrang et al., 2000; Yang et al., 2001; Wang et al., 2019; Zweifel et al., 2015; Chen et al., 2020). This may explain why chemical disinfectants cannot significantly reduce the initial bacterial contamination of the chicken carcass. Combined with the analysis of the process during which feather follicles adsorb bacteria and its structural characteristics, an increase in the permeability of bacterial reduction treatment can solve the problem of bacterial residue adsorbed in the follicle. Furthermore, it has been reported that ultrasound can increase the permeability of disinfectant to muscle tissue and can promote bacterial reduction (Kassem et al., 2018), which means that a combination of ultrasound and disinfectant can reduce bacterial contamination of the feather follicle. Acidification sodium hypochlorite technology can increase the proportion of neutral hypochlorous acid molecules by adjusting the pH of the solution, and the smaller particle size of non-charged hypochlorous acid molecules improves their penetrability of a cell to reduce the bacteria. Therefore, the technique of acidifying sodium hypochlorite can theoretically help control the bacteria absorbed in the follicles.

The role of feather follicles in the contamination of chicken carcasses in the bacterial cross-contamination of chicken skin during processing is still debatable. Berndtson et al. (1992) have demonstrated another possible mechanism of contamination, having isolated *Campylobacter* jejuni from subcutaneous scrapings. On the contrary, Cason et al. (2004) used both featherless and feathered chickens during a bacterial contamination experiment and found no significant difference in the carcass rinse bacterial culture results 30 and 60 s after slaughter. Recently, with the rich development of research methods, there have been a lot of research reports on the bacteria and status in the feather follicle, which will be helpful to evaluate the role of the hair follicle. Chantarapanont et al. (2004) used bacteria transformed with green fluorescent protein plasmid and confocal laser scanning microscopy to find bacteria at depths of 0–30 μm in the folds or follicles of chicken skin, suggesting that the feather follicles may provide adsorption sites and protection against bacterial contamination. Latt et al. (2018) found that 85% of the feather follicles of slaughtered broiler chickens were closed, and 6% were open after chilling. Furthermore, the proportion of enlarged feather follicles has no discernible relationship to the degree of *Campylobacter jejuni* contamination in different areas of the carcass skin (Latt et al., 2018). Based on the analysis of the microstructural changes of chicken skin feather follicles and the changes of the bacteria composition inside the feather follicles above, it is believed that the feather follicle structure inside the chicken skin plays a important role of containing bacterial contamination during the slaughter process, which is not conducive to the cleaning and decontamination of bacteria of chicken carcasses in the slaughter process.

## Conclusion

The closed cavity structure formed in the follicle during defeathering and chilling provides protection for the bacteria adsorbed in the follicle against bacterial reduction measures. Future research should focus on further reducing the residue of bacteria on chicken skin as well as cross-contamination at the source during slaughter while developing technology and measures to eliminate bacterial permeability. The feather follicles could absorb *Acinetobacter* from the contents in the digestive tract, and *Pseudomonas* and *Psychrobacter* originate from the chilling water during the defeathering, evisceration, and chilling processes.

## Data Availability Statement

All raw FASTQ reads used in the study are uploaded to the European Nucleotide Archive (ENA) under accession number PRJEB39877.

## Author Contributions

XZ: study design, manuscript preparation, data acquisition, and analysis. ZP: study design, data acquisition, and analysis. PL, RS, RT, XD, LL, and YZ: data acquisition and analysis. YM: manuscript preparation. XL: conceptualization, data curation, writing – review and editing, and supervision. All authors reviewed the manuscript.

## Conflict of Interest

XZ, RS, RT, XD, LL, and YZ were employed by New Hope Liuhe Co., Ltd.

The remaining authors declare that the research was conducted in the absence of any commercial or financial relationships that could be construed as a potential conflict of interest.
